# Evaluating the Impact of Pontic Geometry on Load to Failure and Displacement in Implant-Supported Monolithic Zirconia Prostheses: An In Vitro Analysis

**DOI:** 10.3390/jfb16030076

**Published:** 2025-02-20

**Authors:** Silvia de la Cruz-Jiménez, Paloma Martínez-Alcaraz, Javier Flores-Fraile, Rubén Agustín-Panadero, Ana Belén Lobo-Galindo, Concepción Carbonell-López, Álvaro Zubizarreta-Macho

**Affiliations:** 1 Faculty of Health Sciences, Alfonso X el Sabio University, 28691 Madrid, Spain; sdelajim@uax.es (S.d.l.C.-J.); palcaraz@uax.es (P.M.-A.); amacho@uax.es (Á.Z.-M.); 2 Department of Surgery, Faculty of Medicine, University of Salamanca, 37008 Salamanca, Spain; alobogal@hotmail.com; 3 Department of Stomatology, Faculty of Medicine and Dentistry, University of Valencia, 46010 Valencia, Spain; rubenagustinpanadero@gmail.com (R.A.-P.); cocarlopez@gmail.com (C.C.-L.)

**Keywords:** implant-supported protheses, pontic, fracture resistance, displacement, monolithic zirconia, bending test

## Abstract

The pontic design may influence the load-to-failure performance of fixed implant-supported screw-retained monolithic zirconia prostheses. This study aimed to evaluate the effect of pontic geometry on the fracture resistance of such restorations. Forty restorations were designed using dental CAD software and divided into four groups (*n* = 10 each): (A) Flat + Wide—pontics with a flat contour, 10 mm in width and 8 mm in height; (B) Concave + Wide—pontics with a concave contour, 10 mm in width and 5.5 mm in height; (C) Flat + Narrow—pontics with a flat contour, 6 mm in width and 8 mm in height; and (D) Concave + Narrow—pontics with a concave contour, 6 mm in width and 5.5 mm in height. All specimens underwent thermal and mechanical cycling, followed by a fracture load test using a three-point bending setup. Maximum fracture loads and displacements were analyzed using one-way ANOVA. Statistically significant differences were observed among the groups for both load to failure (*p* = 0.001) and displacement (*p* = 0.002). These findings indicate that pontic geometry significantly influences the fracture resistance and deformation behavior of monolithic zirconia prostheses.

## 1. Introduction

The field of implantology has evolved significantly in recent years, as dental implants have become the treatment of choice for replacing missing teeth, with predictable results [1]. Currently, patients demand esthetics, as this is associated with youth and beauty, and this desire persists over time due to medical advances and increased life expectancy. Moreover, a natural smile, based on a harmonious combination of teeth, soft tissues, and other oral structures, contributes to attractiveness [2].

The use of implants has shown a success rate of between 96.7% and 100% for single immediate implants, with an average follow-up of 29 months, meaning it is frequently performed as a first-choice treatment [3]. However, the anterior maxillary sector is considered a challenge, as clinicians may encounter peri-implant soft tissue complications, which can lead to unesthetic outcomes [4]. Fortunately, several alternatives exist for replacing lost teeth, including removable prostheses or fixed options such as implants or crowns and bridges on natural teeth. However, dental implants offer advantages that other options do not provide, such as bone maintenance, preservation of adjacent structures, and greater comfort [5,6,7,8].

Nevertheless, complications related to abutments can affect esthetics, with a bluish-gray halo often compromising the overall esthetic outcome. Currently, ceramic materials are preferred for the fabrication of implant abutments [9]. These materials exhibit good resistance to occlusal forces in the anterior region, although less so than titanium abutments. Specifically, zirconium oxide possesses favorable mechanical, optical, and biological properties [10]. As a result, zirconia has become established as one of the most widely used materials in restorative dentistry, particularly for implant-supported prostheses. Zirconia has gained popularity in dental restorations due to its superior mechanical properties, biocompatibility, and esthetics, which closely resemble those of natural teeth. Additionally, its applications range from single crowns to bridges and full-arch frameworks, offering a reliable solution for both natural tooth and implant restorations. As technology advances, with innovations such as CAD/CAM and 3D printing, the precision and efficiency of zirconia restorations have improved, although the long-term longevity of these treatments remains under evaluation [11]. Moreover, the fabrication of these structures, whether zirconia or metal, has transitioned into a digital process. Specifically, esthetic prostheses on implants using zirconia are designed and manufactured digitally with dental CAD/CAM technology, ensuring prostheses that are both aesthetically and functionally precise while minimizing errors in a highly predictable manner [12].

Furthermore, zirconia is becoming an increasingly popular restorative material in restorative dentistry [13] due to its esthetic potential, natural appearance, high resistance, white color, translucency, biocompatibility, and suitability for patients allergic to metal [14]. Simple contact tests can also be performed to evaluate the mechanical integrity of zirconia dental ceramics, assessing hardness, identifying critical variations in material properties, and quantifying damage in different crown areas [15]. Additionally, zirconia crowns are preferable to stainless-steel crowns in terms of gingival health; primary posterior teeth restored with zirconia crowns have demonstrated better gingival health and a 52% lower risk of clinical failure compared to those restored with stainless-steel crowns [16].

At room temperature, zirconia exists in its monoclinic phase. However, when heated to 1170 °C or exposed to low-temperature degradation (LTD) conditions, it transforms into the tetragonal phase [17]. With further temperature increases to 2370 °C, or under processes such as aging or hydrothermal degradation, the material progressively reverts to the monoclinic phase.

Achieving a strong bond between the zirconia restoration and the tooth is crucial for the longevity of the prosthetic restoration [18,19]. To ensure proper adhesion with the luting cement, zirconia requires surface treatment, typically through acid etching, to create surface roughness and enhance bonding [20].

There is substantial background that underscores the importance of fracture resistance and displacement in implant-supported prostheses. Given the occlusal forces exerted on prostheses during mastication, the ability of zirconia to withstand these mechanical stresses without fracturing is critical for the long-term success of restorations. The fracture resistance of zirconia has been extensively studied, and while it offers superior strength compared with other ceramics, ensuring the optimal performance under varying clinical conditions remains a key concern in prosthodontics. Specifically, zirconia is considered the hardest among the various restorative materials used in dentistry [17]. Its flexural strength and hardness far exceed those of other materials employed in dental restorations. Conventional zirconia also demonstrates superior biaxial flexural strength compared with high-translucent monolithic zirconia [20]. Moreover, the fracture toughness of 5Yttria (Y)-tetragonal zirconia polycrystal (TZP) (5 mol% Y-TZP) is approximately 50% lower than that of 3Y-TZP (3 mol% Y-TZP), primarily due to the higher yttria content, which increases the cubic phase proportion [21]. According to a recent study by Liao et al. (2023), the flexural strength values were 584 (±158) MPa for 3Y-TZP and 373 (±104) MPa for 5Y-TZP [22]. However, a more in-depth analysis of the mechanical aspects, challenges, and considerations related to zirconia-based fixed dental prostheses is recommended. Factors such as the material’s resistance to tensile and flexural stress, the potential chipping of the veneering ceramic, and the effects of connector design and thickness on the overall prosthesis strength are crucial in determining the long-term success of these restorations. Additionally, ensuring the fit and stability of zirconia prostheses on implant abutments is essential to prevent micromovements and avoid mechanical complications, further highlighting the need for a comprehensive understanding of these parameters.

Although fully ceramic crowns offer excellent biocompatibility and aesthetic appearance, they may be more prone to fracture. Additionally, the mechanical properties of monolithic zirconia as a restorative material are superior, potentially preventing fractures caused by chewing hard foods [23]. Moreover, monolithic zirconia crowns have shown no detectable adverse effects on periodontal tissues, and wear on opposing teeth is minimal, demonstrating good potential for short-term clinical application in posterior tooth restorations [24]. It is also important to evaluate whether surface treatments and cement selection for traditional zirconia or lithium disilicate crowns influence their fracture load. Another crucial aspect is assessing the clinical and radiographic effectiveness of zirconia crowns compared to stainless-steel crowns in the rehabilitation of primary posterior teeth [25,26].

Pjetursson et al. conducted a systematic review and meta-analysis to evaluate and compare the 5- and 10-year survival rates of various tooth-supported and implant-supported fixed dental prostheses (FDPs) and single crowns (SCs), as well as to identify the occurrence of biological and technical complications. They reported a 5-year survival rate of 93.8% for conventional tooth-supported FDPs, 95.2% for implant-supported FDPs, and 94.5% for implant-supported SCs. After 10 years, the survival rate decreased to 89.2% for conventional FDPs, 86.7% for implant-supported FDPs, and 89.4% for implant-supported SCs.

Despite the high survival rates, 38.7% of patients with implant-supported FDPs experienced complications after 5 years, compared to 15.7% for conventional FDPs. Biological issues, such as caries and pulp vitality loss, were the most common complications in conventional tooth-supported FDPs. Technical complications were more frequent in implant-supported FDPs and included fractures of veneering materials (e.g., ceramic fractures or chipping), abutment or screw loosening, and retention loss [27]. Additionally, in a study using the finite element method, Chander et al. found no statistically significant differences in stress distribution between tooth-supported and implant-supported FDPs when considering various connector widths, cuspal inclinations, and force angulations [28]. However, Luft et al. reported that the cross-sectional geometry of the connector significantly influenced the mechanical fatigue resistance of implant-supported fixed partial prostheses made of monolithic zirconia [29].

The aim of this study was to evaluate and compare the influences of the geometric designs of pontics on the load to failure and displacement of fixed, implant-supported, screw-retained monolithic zirconia prostheses. The null hypothesis (H0) stated that there would be no differences in load to failure and displacement based on the geometric design of the pontics in fixed, implant-supported, screw-retained monolithic zirconia prostheses.

## 2. Material and Methods

### 2.1. Study Design

A randomized in vitro study was conducted at the Department of Surgery, University Alfonso X el Sabio (Madrid, Spain), and the Faculty of Medicine and Dentistry, University of Valencia (Valencia, Spain), between September 2022 and October 2023. Moreover, this study received approval from the Ethics Committee of the Faculty of Health Sciences, University Alfonso X el Sabio (Madrid, Spain), in October 2022 (Process No. 18/2022).

### 2.2. Experimental Procedure

Eighty dental implants with a diameter of 4.6 mm and a length of 12 mm, featuring conical walls and an internal taper (Ref.: TLX4612, BioHorizons, Birmingham, AL, USA), were embedded in pairs into an epoxy resin material (Exakto-Form^®^, Bredent, Senden, Germany) to simulate the mechanical loading properties of bone tissue (flexural strength [DIN 53452]: 50 N/mm^2^; E-modulus [DIN 53452]: 3900 N/mm^2^). The operator was required to place the dental implants (Ref.: TLX4612, BioHorizons, Birmingham, AL, USA) with a 6 mm inter-implant distance [30], using a positioning template to ensure parallelism between each pair of dental implants and to standardize the insertion depth into the epoxy resin material (Exakto-Form^®^, Bredent, Senden, Germany). Subsequently, digital impressions were taken using an intraoral scanner (True Definition, 3M ESPE™, Saint Paul, MN, USA) after placing scan bodies (Ref.: 80610156, BioHorizons, Birmingham, AL, USA).

**Figure 1:**
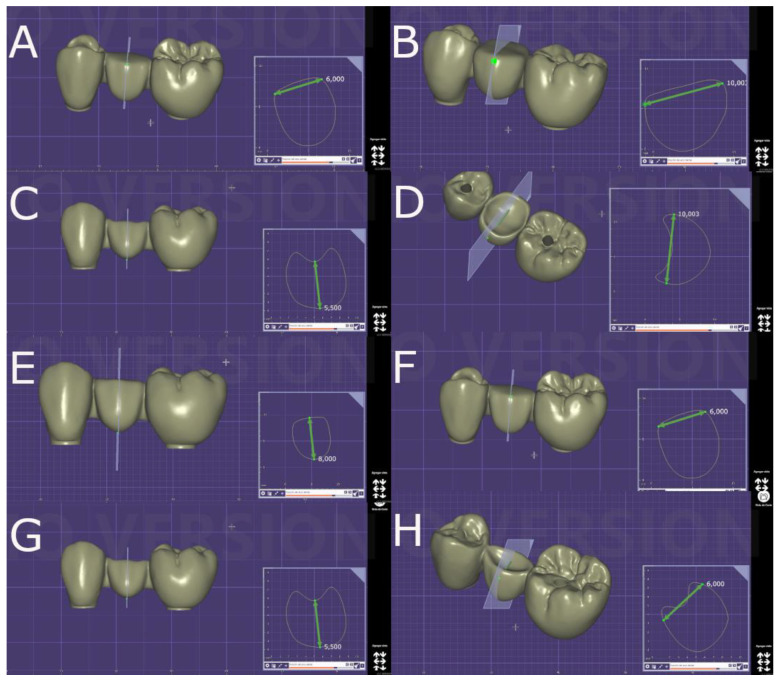
(**A**) Buccal and (**B**) occlusal views of the geometric design of the pontic of the fixed implant-supported and screw-retained monolithic zirconia restorations with 10 mm buccolingual width and 8 mm height with a flat occlusal surface. (**C**) Buccal and (**D**) occlusal views of the geometric design of the pontic of the fixed implant-supported and screw-retained monolithic zirconia restorations with 10 mm buccolingual width and 5.5 mm height with a flat occlusal surface. (**E**) Buccal and (**F**) occlusal views of the geometric design of the pontic of the fixed implant-supported and screw-retained monolithic zirconia restorations with 6 mm buccolingual width and 8 mm height with a flat occlusal surface. (**G**) Buccal and (**H**) occlusal views of the geometric design of the pontic of the fixed implant-supported and screw-retained monolithic zirconia restorations with 6 mm buccolingual width and 5.5 mm height with a flat occlusal surface.

**Figure 2:**
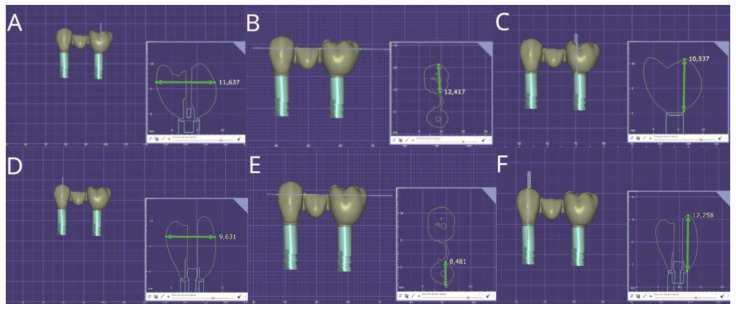
(**A**) Buccal view of the buccolingual, (**B**) mesio-distal, and (**C**) occluso-cervical dimensions of the molar teeth, and (**D**) buccal view of the buccolingual, (**E**) mesio-distal, and (**F**) occuso-cervical dimensions of the premolar teeth of the fixed implant-supported and screw-retained monolithic zirconia restorations.

Afterwards, forty fixed, implant-supported, screw-retained monolithic zirconia restorations (Lava™ Esthetic, 3M™, Saint Paul, MN, USA) were digitally designed using dental planning software (EXOCAD 3.1, Darmstadt, Germany), simulating four different pontic designs (Figure 1A–H) in a prosthetic framework corresponding to tooth positions 1.4 to 1.6 (Figure 2A–F).

The geometric designs of the pontic of the fixed implant-supported and screw-retained monolithic zirconia restorations (Lava™ Esthetic, 3M™, MN, USA) were randomly distributed (Epidat 4.1, Galicia, Spain) into the following study groups: Group A: buccolingual width: 10 mm and height: 8 mm with a flat occlusal surface (*n* = 10) (Flat + Wide); group B: buccolingual width: 10 mm and height: 5.5 mm with a concave occlusal surface (*n* = 10) (Concave + Wide); group C: buccolingual width: 6 mm and height: 8 mm with flat occlusal surface (*n* = 10) (Flat + Narrow), and group D: buccolingual width: 6 mm and height: 5.5 mm with a concave occlusal surface (*n* = 10) (Concave + Narrow).

Next, the fixed implant-supported and screw-retained monolithic zirconia restorations (Lava™ Esthetic, 3M™, MN, USA) were manufactured using computer-aided design/computer-aided manufacturing (CAD-CAM) with a titanium base abutment measuring 4.5 mm in height (Ref. PGHYBN, BioHorizons, Birmingham, AL, USA). Specifically, the fixed implant-supported and screw-retained monolithic zirconia restorations were milled (VHF S 5) using a bit sequence as follows: ball diameters 2 × 17 and 1 × 17, flat milling cutter diameter 1.2 × 15, and ball diameter 0.6 × 8. Afterwards, all zirconia restorations were sintered in a program that completes 3 to 6 pieces in 676 min with a temperature increase in ramps, as recommended by the manufacturer, up to a maximum temperature of 1550 °C with slow cooling and, finally, glazing. The monolithic zirconia restorations (Lava™ Esthetic, 3M™, MN, USA) were screwed in place according to the manufacturer’s recommended torque load (30 Ncm) using a digital torque gauge (BTGE-G, Tohnichi America, Buffalo Grove, IL, USA).

### 2.3. Thermal and Mechanical Cycling Fatigue

The passive fit of the prosthesis was analyzed before mechanical testing by a single operator (Á.Z.-M.). The experimental groups underwent both thermal and mechanical cycling. The restorations were subjected to thermal fatigue using a Thermocycling TC-3 device (SD Mechatronik, Feldkirchen-Westerham, Germany), and were immersed in distilled water for 43 cycles per hour over the course of one day, totaling 6000 thermal cycles. The temperature was varied between 5 and 55 °C, with a 30-second dwell time for each cycle [31].

Mechanical cycling was induced using a masticatory simulator (SD Mechatronik, Masticatory Simulator CS-4, Mechatronik GmbH, Feldkirchen, Germany), which applied a load of 80 N [32] for 240,000 masticatory cycles. The load was applied axially to the center of the occlusal surface of the pontic, with a vertical displacement of 2 mm at a frequency of 2 Hz and a speed of 40 mm/s, using a point of contact [33].

### 2.4. Fracture Load Test

**Figure 3:**
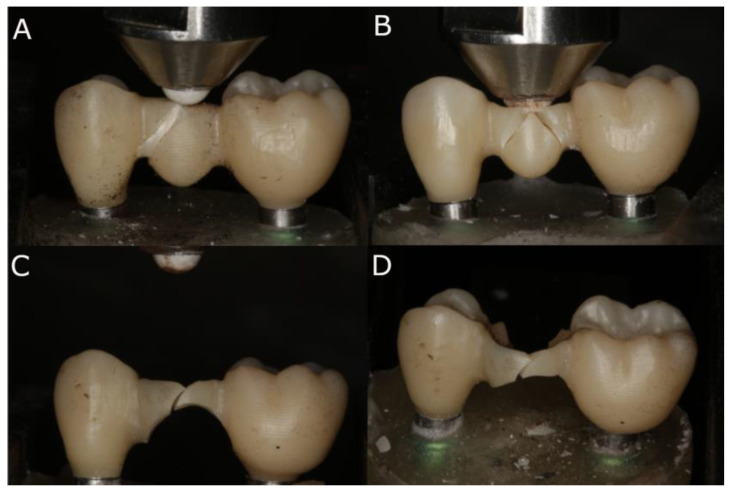
(**A**) Flat + Wide, (**B**) Concave + Wide, (**C**) Flat + Narrow, and (**D**) Concave + Narrow fixed implant-supported and screw-retained monolithic zirconia restorations fractured by a static load after the bending test.

After the fatigue simulation, all specimens underwent a bending test until fracture using a universal testing machine (UTM) (Shimadzu^®^ AG-100 KN, Shimadzu Corporation, Kyoto, Japan) at a crosshead speed of 0.5 mm/s (ME 405/10, SERVOSIS, Madrid, Spain). The machine was equipped with a 5000 N load cell and operated at room temperature (23 ± 1 °C). The force was applied vertically downward, perpendicular to the occlusal plane, simulating masticatory forces. Axial compressive loads were applied using a cone-shaped stainless-steel bar with a rounded tip, which was attached to the UTM. Specifically, the stainless-steel bar had a diameter of 1 mm and exerted a point contact of 1 mm on the contact surface of the samples, while the alumina ball of the static machine had a diameter of 4 mm. This customized load piston was placed perpendicularly at the center of the occlusal surface, making contact only with the restorative material, until the specimen fractured (Figure 3A,D). Fracture was identified by a sharp decrease in the stress plot. The force applicator, an aluminum ball, was positioned at the center of the occlusal surface of the pontic. The results were recorded using the inbuilt software (PCD2K, SERVOSIS 1.0), and force (N)–displacement (mm) curves were automatically generated. The maximum force (N) and maximum displacement (mm) sustained by the specimens until fracture occurred were recorded [34].

### 2.5. Statistical Tests

Forty fixed implant-supported and screw-retained monolithic zirconia restorations were included in this study to ensure a power effect of 0.8 for detecting statistically significant differences. Student’s t-test of two independent samples was used to evaluate the null hypothesis H₀: μ₁ = μ₂, with a significance level of 0.05.

A table was generated with the summary statistics for each response variable according to the group: number of observations, mean, median, standard deviation, and the minimum and maximum values. These were graphically represented using a box plot.

Normality tests were performed using the Shapiro–Wilk test. For variables with a normal distribution, the comparison of means between groups was performed using analysis of variance (ANOVA). If statistically significant differences between groups were detected, pairwise comparisons were performed, and *p*-values were adjusted using the Tukey method to correct for type I error. Statistical analysis was performed using SAS software v9.4 (SAS Institute Inc., Cary, NC, USA). Statistical decisions were based on a significance level of 0.05.

## 3. Results

### 3.1. Load to Failure

Normality tests of the load-to-failure (N) values were performed using the Shapiro–Wilk test (Table 1).

The means and SD values for the load to failure (N) according to the geometric design of the pontics of fixed implant-supported and screw-retained monolithic zirconia prostheses are displayed in Table 2.

**Figure 4:**
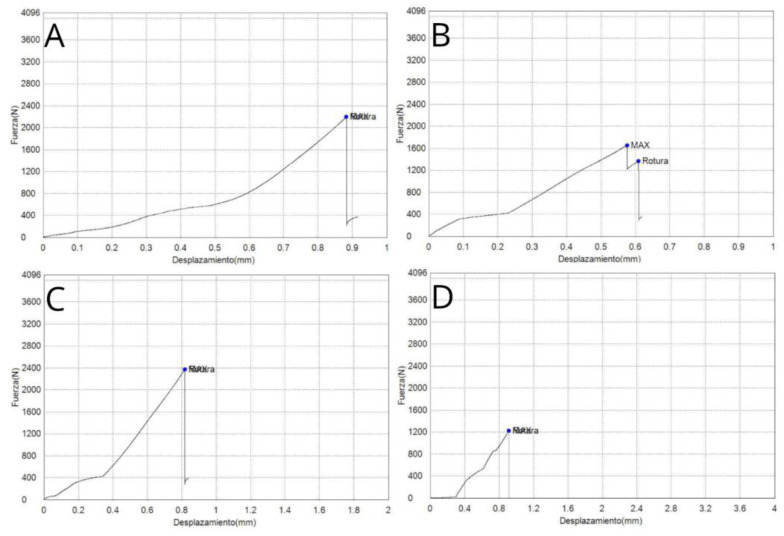
Force–displacement curves of (**A**) Flat + Wide, (**B**) Concave + Wide, (**C**) Flat + Narrow, and (**D**) Concave + Narrow fixed implant-supported and screw-retained monolithic zirconia restorations.

ANOVA showed statistically significant differences (*p* = 0.001) between the load to failure (N) of Concave + Wide (2217.09 ± 129.40 N), Flat + Narrow (1458.33 ± 798.13 N), Flat + Wide (2259.75 ± 519.70 N), and Concave + Narrow (1486.56 ± 397.26 N) geometric designs of the pontics of fixed implant-supported and screw-retained monolithic zirconia prostheses (Table 1 and Figure 4).

Differences were found between the two wide groups (higher values) and the two narrow groups (lower values).

**Figure 5:**
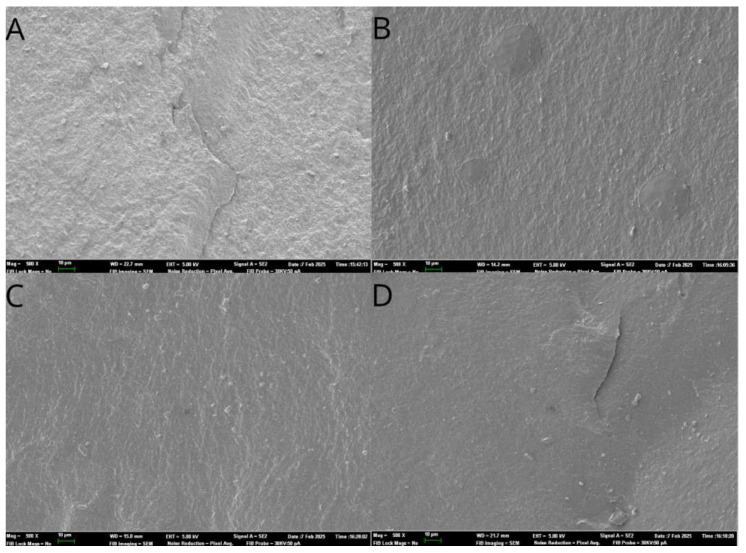
A fractographic analysis of the (**A**) Flat + Wide, (**B**) Concave + Wide, (**C**) Flat + Narrow, and (**D**) Concave + Narrow fixed implant-supported and screw-retained monolithic zirconia restorations.

Additionally, fractographic analysis was performed with a scanning electron microscope (Phenom XL Desktop SEM, Thermo Fisher Scientific, Waltham, MA, USA, Figure 5).

### 3.2. Displacement Resistance

Normality tests of the displacement (mm) values were performed using the Shapiro–Wilk test (Table 3).

**Figure 6:**
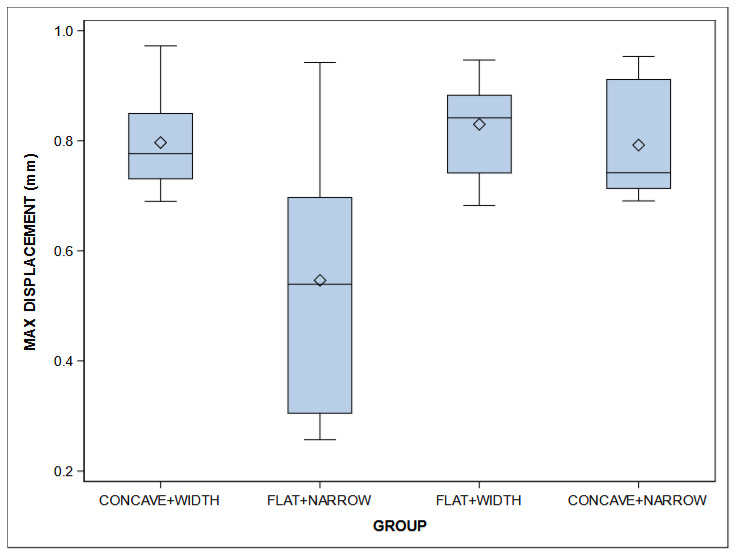
Box plots of the displacement (mm) according to the geometric design of the pontics of fixed implant-supported and screw-retained monolithic zirconia prostheses. The horizontal line in each box represents the median value.

The means and SD values for the displacement (mm) according to the geometric design of the pontics of fixed implant-supported and screw-retained monolithic zirconia prostheses are displayed in Table 4 and Figure 6.

ANOVA showed statistically significant differences (*p* = 0.002) between the displacement (mm) of Concave + Wide (0.80 ± 0.09 mm), Flat + Narrow (0.55 ± 0.25 mm), Flat + Wide (0.83 ± 0.09 mm), and Concave + Narrow (0.79 ± 0.10 mm) geometric designs of the pontics of fixed implant-supported and screw-retained monolithic zirconia prostheses, especially between Concave + Narrow and Flat + Narrow (*p* = 0.0014), between Concave + Wide and Flat + Narrow (*p* = 0.0018), and between Flat + Narrow and Flat + Wide (*p* = 0.0003) (Figure 3).

Briefly, the geometric design of the Flat + Wide pontics demonstrated a superior performance compared to the Concave + Wide, Concave + Narrow, and Flat + Narrow designs, based on the displacement (mm) of fixed implant-supported and screw-retained monolithic zirconia prostheses.

## 4. Discussion

The results presented in this study allowed us to reject the null hypothesis (H₀), which stated that the geometric design of the pontics would produce no differences in the load to failure and displacement of fixed implant-supported and screw-retained monolithic zirconia prostheses.

This study showed that the differences in load to failure among the Concave + Wide (2217.09 ± 129.40 N), Flat + Narrow (195,768.33 ± 30,291.62 N), Flat + Wide (2259.75 ± 519.70 N), and Concave + Narrow (97,467.38 ± 303,361.94 N) geometric designs of pontics for fixed implant-supported and screw-retained monolithic zirconia prostheses were not statistically significant. On the contrary, Luft et al. assessed the load-bearing capacity under fatigue of implant-supported fixed partial prostheses made of monolithic zirconia with various connector cross-sectional geometries (round, square with rounded angles, or trapezoidal with rounded angles). They reported that the geometry of the connector cross-section significantly impacted the mechanical fatigue performance of implant-supported fixed partial prostheses made from monolithic zirconia, concluding that the connector cross-sectional geometry significantly affects the mechanical fatigue resistance of fixed implant-supported monolithic zirconia prostheses, especially round connectors [34]. More in line with our findings, Muhsin et al. conducted a finite element study to analyze the stress–deformation behavior of a metal, fixed, partial denture pontic under different loads, using two types of connectors. They found that the stress and deformation patterns on the pontic occlusal surface were similar under various loads, regardless of whether square or round connectors were used. Yet, they observed that, with the same connector designs, the pontic occlusal surface experienced more significant deformation at three specific loaded points compared to each individual point [35]. In the present study, all the fixed implant-supported prostheses were designed and manufactured with a similar anatomical design (including connectors), except for the geometric design of the pontics. Therefore, the differences in fracture resistance among the study groups were not statistically significant.

The parameters used in the thermal and mechanical cycling fatigue procedure were established according to ISO standard parameters [34] and based on previous studies, which included artificial aging procedures (cyclic loading and thermocycling) to reproduce the conditions to which restorations are subjected in the oral environment (mechanical stress and temperature changes) [33,36,37,38,39,40,41,42,43,44,45,46,47,48]. Most suggest that in vitro studies should include fatigue procedures to analyze the fracture resistance of new ceramic materials, as these may experience a decrease in fracture resistance due to degradation from the repeated application of thermal stress on prosthetic restorations [37,41,42].

Kohorst and Iljima reported decreases in fracture resistance of 40% and 54–64%, respectively, when compared to the static load of zirconia restorations or abutments [45,49]. The applied load was 80 N, which is similar to the force used by Rosentritt [50] and represents a conventional occlusal force. This load is within the range of forces recorded clinically, which vary between 12 and 70 N. The clinical relevance of applying lower physiological forces (30–49 N) is questionable, as forces applied to incisors range from 40 to 370 N. Studies using lower force intensities [38,45,50,51] may not provide results directly applicable to daily clinical practice.

In 2015, Gehrke conducted a study using the same machine to test the fatigue resistance and thermal cycling as the one in our study. In their research, they subjected all specimens to dynamic loading for 120,000 cycles, applying 100 N of force about 2 mm below the incisal edge, as well as 1000 thermal cycles. All specimens survived the dynamic fatigue phase without any fractures or delaminations in the crowns [49].

The displacement recorded by the customized load piston of the universal testing machine was assumed to result from the deflection of the fixed implant-supported and screw-retained monolithic zirconia prostheses. This parameter can be used to measure the elastic modulus of the materials [52], and Mahmood et al. reported that the fracture load of fixed implant-supported restorations can be influenced by the elastic modulus of the abutment [53]. Scherrer and Rijk suggested that the fracture resistance of all-ceramic crowns decreases with higher values of elastic modulus [54], as a higher elastic modulus indicates a stiffer material, which deforms less when subjected to a given force.

This relationship between load to failure and displacement was confirmed in the present study. The Flat + Narrow geometric design of the pontics exhibited the lowest values for load to fracture (1458.33 ± 798.13 N) and displacement (0.55 ± 0.25 mm), followed by Concave + Narrow (1486.56 ± 397.26 N; 0.79 ± 0.10 mm), Concave + Wide (2217.09 ± 129.40 N; 0.79 ± 0.10 mm), and Flat + Wide (2259.75 ± 519.70 N; 0.79 ± 0.10 mm).

Previous studies related to the fracture resistance of implant-supported restorations manufactured by CAD-CAM procedures have highlighted the influence of the connector design on the framework. Hadzhigaev et al. suggested that the distal connector is the weakest area of a three-unit, all-ceramic fixed prosthesis in the posterior region [55]. In line with this, the visual fractographic analysis conducted in the present study revealed that the majority of fracture patterns across all study groups were related to the connectors of the pontic. Additionally, the present study also assessed the displacement of the prostheses. According to Ko et al., displacement from loading can generate high stress in the margin area of the prosthesis, potentially leading to fracture and failure [56]. This underscores the importance of analyzing displacement in relation to the pontic design. Even with high-stiffness materials like monolithic zirconia, axial displacement could not be entirely prevented.

Furthermore, Gehrke et al. suggested that clinicians should be mindful of the interocclusal space when designing implant-supported restorations, as a reduced space can influence the prosthetic design, potentially leading to complications such as fracture or decementation of coated zirconia crowns [57]. However, they did not report a significant decrease in fracture resistance when the space for the prosthetic restoration was reduced [58]. Similarly, in the present study, the concave restorations did not show a significant decrease in fracture resistance.

While CAD/CAM technology and 3D printing have improved the precision and efficiency of dental prostheses fabrication, there is still no conclusive evidence demonstrating a significant increase in the long-term survival of these treatments compared to traditional methods [59]. For this reason, we designed a prosthetic framework simulating tooth positions 1.4 to 1.6. Previous long-term studies have shown that prosthetic restorations made with tetragonal yttria-stabilized zirconia and feldspathic ceramic coatings achieved good durability results, particularly in the posterior region [60,61,62]. Additionally, Refaie et al. reported that implant-supported restorations manufactured using monolithic zirconia and titanium abutments exhibited high fracture resistance in posterior regions [63]. Therefore, monolithic zirconia was chosen as the material for this study.

Fixed and partial implant-supported monolithic zirconia restorations have demonstrated predictable short-term survival rates (up to 5 years); however, there is insufficient evidence regarding their medium-term survival (>5 years). Moreover, monolithic zirconia restorations—both anterior and posterior—have shown a lower fracture rate compared to layered restorations [64,65].

This study provides novel insights obtained by investigating the specific impact of pontic geometric design on the fracture resistance and displacement of fixed, implant-supported, screw-retained monolithic zirconia prostheses, an area that has not been fully explored in previous research. Unlike prior studies, this research compares different pontic designs with varying widths and occlusal surfaces, offering a more comprehensive understanding of how these geometric variations influence the mechanical behavior of zirconia prostheses. By analyzing both fracture resistance and displacement under simulated masticatory conditions, this study adds valuable data on the biomechanical performance of monolithic zirconia restorations, which is crucial for optimizing prosthesis design in clinical practice. The study introduces a new angle by focusing on the displacement of the pontic designs before fracture occurs, providing evidence that the geometric configuration can significantly affect the overall performance of implant-supported zirconia prostheses. In doing so, this research expands the current literature by highlighting that although fracture resistance may not vary significantly, the displacement behavior of different pontic designs can differ substantially, which could inform future design choices for implant-supported prostheses.

This study has certain limitations typical of in vitro research on the material resistance of implant prostheses. Specifically, replicating all patient-related factors that can affect the prosthesis is challenging, making it difficult to directly apply the findings to real-world clinical scenarios. Future investigations could benefit from comparing monolithic zirconia with other materials featuring similar geometric designs on conventional implants, such as tissue-level implants (with a divergent transmucosal neck) and bone-level implants (with convergent abutments, both with and without prosthetic finish lines). This would help determine whether the type of restorative material or the implant design influences the mechanical behavior of the prosthesis–abutment–implant complex. Additionally, exploring the impact of connector dimensions and shape on biomechanical stress, as well as testing other materials that can better absorb the load without transmitting excessive stress to the attachments, is of great interest.

## 5. Conclusions

The results show that the geometric design of the pontics affects both the load to failure and displacement of fixed implant-supported and screw-retained monolithic zirconia prostheses. Chiefly, the Concave + Wide geometric design of pontics significantly influences the displacement of these prostheses.

## Figures and Tables

**Table 1:** Test for normality for the load-to-failure (N) values.

Tests for Normality				
Test	Statistic		*p*-Value	
Shapiro–Wilk	W	0.98	*p*	0.719

**Table 2:** Descriptive statistics of the load to failure (N) according to the geometric design of the pontics of fixed implant-supported and screw-retained monolithic zirconia prostheses.

Study Group	*n*	Mean	SD	Minimum	Maximum
Concave + Wide	10	2217.09	129.40	2003.53	2417.63
Flat + Narrow	10	1458.33	798.13	521.28	2376.41
Flat + Wide	10	2259.75	519.70	1539.07	3281.16
Concave + Narrow	10	1486.56	397.26	960.86	2015.05

SD: standard deviation.

**Table 3:** Test for normality for the displacement (mm) values.

Tests for Normality				
Test	Statistic		*p*-Value	
Shapiro–Wilk	W	0.96	*p*	0.242

**Table 4:** Descriptive statistics of the displacement (mm) according to the geometric design of the pontics of fixed implant-supported and screw-retained monolithic zirconia prostheses.

Study Group	*n*	Mean	SD	Minimum	Maximum
Concave + Wide	10	0.80	0.09	0.69	0.97
Flat + Narrow	10	0.55	0.25	0.26	0.94
Flat + Wide	10	0.83	0.09	0.68	0.95
Concave + Narrow	10	0.79	0.10	0.69	0.95

SD: standard deviation.

## Data Availability

The data presented in this study are available on request from the corresponding author due to the policy of the research team.
